# Tools and techniques for classifying behaviours in canine epilepsy

**DOI:** 10.3389/fvets.2023.1211515

**Published:** 2023-11-01

**Authors:** Emily Folkard, Lee Niel, Luis Gaitero, Fiona May Kier James

**Affiliations:** ^1^Department of Clinical Studies, University of Guelph, Guelph, ON, Canada; ^2^Department of Population Medicine, University of Guelph, Guelph, ON, Canada

**Keywords:** canine epilepsy, idiopathic epilepsy, behavioural comorbidities, questionnaires, electroencephalography, actigraphy

## Abstract

Idiopathic epilepsy is the most common neurological disease in dogs. Similar to humans, dogs with epilepsy often experience behavioural comorbidities such as increased fear, anxiety, and aggression, as reported by their caregivers. Investigations of behaviour in canine epilepsy have yet to untangle interictal and pre and postictal behaviours, prodromal changes, and seizure-precipitating factors. Under-recognition of absence and focal seizures further complicates these assessments. These complex behavioural presentations in combination with caring for an epileptic animal have a significant negative impact on the dog’s and caregiver’s quality of life. Despite the growing recognition of behavioural comorbidities and their impact on quality of life in dogs with epilepsy, few objective research methods for classifying and quantifying canine behaviour exist. This narrative review examines the strengths, limitations, and granularity of three tools used in the investigation of canine behaviour and epilepsy; questionnaires, electroencephalography, and actigraphy. It suggests that a prospective combination of these three tools has the potential to offer improvements to the objective classification and quantification of canine behaviour in epilepsy.

## 1. Introduction

Epilepsy is the most common neurological disease that dogs present with in veterinary medicine. As a subtype, idiopathic epilepsy (IE, defined as reoccurring seizures in the absence of any identifiable etiopathogenesis other than a suspected genetic one) affects the largest proportion of the canine population at approximately 0.5–1% (1). Epilepsy is also a common neurological disease in people, where psychosocial comorbidities are a well-recognized phenomenon. People with epilepsy have been found to have a higher prevalence of comorbid psychiatric disorders such as depression, anxiety, and cognitive dysfunction, in comparison to individuals with other neurological diseases (2, 3). Recent studies recognize the existence of similar comorbidities in dogs with epilepsy. Normal behaviours become comorbid conditions in dogs when the behaviour becomes more frequent or extreme without environmental justification and is uncontrollable by the dog (4). Dogs with IE have a higher prevalence of comorbid behavioural conditions such as fear, anxiety, and aggression in comparison to neurotypical dogs according to caregiver-completed surveys (5, 6). Caregivers often report increased anxiety in their dog as both a pre-ictal and interictal behaviour (5–7).

In addition to pathologic behavioural changes in canine epilepsy, the use of anti-seizure drugs (ASDs) has an impact on canine behaviour. Currently the first line of treatment in canine epilepsy, ASDs are not successful in significantly reducing seizure frequency in approximately 33% of dogs (8). Seizures in uncontrolled animals are seemingly unpredictable, leaving caregivers in a constant state of stress waiting for the next occurrence, and resulting in considerable disturbance to their everyday lives. ASD usage has commonly reported adverse effects including lethargy, lack of coordination, polyuria, and polydipsia (8, 9). Persistent seizures and ASD adverse effects both have a significant negative impact on the dog and caregiver’s QoL (10). The combination of behavioural comorbidities and ASD-related behavioural changes contributes significantly to the difficulty of caring for a dog with epilepsy and negatively affects the dogs’ and caregivers’ quality of life (QoL) (10, 11). Despite the similar increase in the prevalence of behavioural comorbidities seen in people and dogs with epilepsy, research regarding neurobehavioural comorbidities in canine epilepsy is limited. Therefore, there is an urgent need to identify clinical tools that are practical and effective for prognostication, more specifically, the prediction of seizures and response to therapy. If caregivers can predict seizure occurrence, they can administer ASD treatment prior to a seizure to decrease its severity or prevent it from happening altogether. To improve dog and caregiver QoL, further research investigating neurobehavioural comorbidities in canine epilepsy is essential.

Veterinary medicine needs greater clarity in the characterization of canine behaviours around seizures, particularly differentiating interictal, pre- or postictal changes, and psychosocial comorbidities. Psychosocial comorbidities, such as anxiety and aggression, occur as a result of psychological and environmental factors and alter physical and cognitive functioning. Interictal changes in behaviour are classified as behaviours that occur in the period between seizures, whereas pre- and post- ictal behaviours occur immediately before and after seizures, respectively. Certain seizure types, such as absence and focal, further complicate behaviour characterization around seizures. Absence seizures are characterized by a brief period of unresponsiveness and blank staring that may be accompanied by myoclonus, whereas focal seizures are characterized by behaviours such as head and facial twitching, lip smacking, and excessive blinking (12, 13). The paroxysmal physical presentations associated with absence and focal seizures can be misinterpreted as abnormal behaviours such as compulsive tendencies, sleep disorders, or as movement disorders (non-epileptic transient, involuntary, and repetitive or tonic skeletal muscle contractions or movements). This overlap between the physical manifestations exhibited in absence or focal seizures and movement disorders increases the complexity of canine seizure behaviour classification.

Changes in canine behaviour can be investigated at a variety of time intervals prior to and following the onset of IE, adding yet another level of complexity to behaviour characterization. Behavioural comparisons can be made over a large timespan such as pre-to post-diagnosis, or a more granular approach can be taken by comparing behaviours over a shorter timespan such as pre- to post-ictal or interictal. Thus, it is important to keep in mind what questions are being asked and which behaviours are to be investigated when determining the time window and method of comparison.

Recent early progress made by various international groups has advanced our understanding of canine behaviour surrounding seizures and IE. Methods employed to investigate seizures and associated behaviours include electroencephalography (EEG), survey tools, and actigraphy, among others discussed in previously published literature (14). EEG uses electrodes to record electrical activity in the brain to determine the presence of seizures, and epileptogenic ictal (within a seizure) or interictal (between seizures) discharges (15). Survey tools, such as validated questionnaires, have been used to investigate both seizure characteristics, like seizure triggers or frequency, as well as subjective caregiver-reported behavioural changes (16, 17). Actigraphy measures physical activity and can be used to detect specific behaviours by sensing movement in three axes and has also been used to investigate generalized tonic–clonic (GTC) seizures in dogs (18). Strengths and limitations exist for each of these methods.

A comprehensive approach to collecting objective behavioural data in dogs with epilepsy is essential to continue to increase our understanding of these behavioural comorbidities. This data will feed more effective clinical tools for epilepsy diagnosis and treatment, and aid in developing an approach for seizure prediction by identifying reliable behavioural changes that occur pre-ictally. The ability for clinicians and caregivers to predict seizure occurrence would drastically improve the QoL of dogs with epilepsy and their caregivers that are negatively affected by behavioural comorbidities and ASD adverse effects (9–11). Given the growing recognition and importance of this intersection, this narrative review takes a unique approach to better understand epileptic canine behaviour by highlighting the current tools used in veterinary research for investigating behaviour and seizure characteristics in dogs and suggests a potential method for collecting objective behavioural data as a step towards seizure prediction abilities.

## 2. Tools for investigating canine behaviour

### 2.1. Questionnaires

Questionnaires are tools commonly employed in research to gather detailed dog and household information from owners. A range of questionnaires have been developed to investigate both the general behaviour of dogs, as well as behavioural changes that occur because of health issues, including behavioural comorbidities in diseases such as epilepsy. In canine epilepsy, questionnaires have been used to gain insight into various epilepsy phenomena including seizure frequency and intensity, ASD adverse effects, QoL concerns, and behavioural comorbidities, as well as the caregiver’s perspective (6, 9, 11, 19–21). Questionnaires allow examination of these phenomena at different time intervals following the onset of IE, ranging from granular periods such as immediately pre- to post-seizure to broader periods such as pre- to post-diagnosis. It is important to consider the time scope of available validated questionnaires when implementing them in research, as some are better suited for more fine-grained versus broad behavioural investigations. For this reason, some researchers elect to create their own questionnaires or combine theirs with previously validated ones depending on their research goals and the period under investigation. For example, in 2011, a group of researchers from the United Kingdom (UK) sought to investigate potential behavioural changes seen in dogs with IE pre- and post-diagnosis by combining two questionnaires previously validated for separate studies (19). Completed by the caregivers of dogs with IE, the tool captured the dog’s medical history and the occurrence and severity of the dog’s behavioural problems over recent months. They found a significant increase in post-epilepsy onset scores for fear, anxiety, aggression, abnormal reactivity, attachment disorder, and abnormal perception versus the pre-epilepsy scores as perceived by the caregivers. The caregivers further believed that at least one behaviour changed significantly from pre- to post-epilepsy onset in 71% of the 80 participating dogs, supporting the idea that caregivers commonly perceive behavioural comorbidities in canine epilepsy (19). Thus, the careful selection and combination of existing validated questionnaires can generate behavioural insights.

Of the questionnaires developed and validified specifically to help assess the many facets of behaviour in dogs, a notable one is the Canine Behavioural Assessment and Research Questionnaire (CBARQ) (16). CBARQ is commonly used by clinicians and researchers to better understand a dog’s temperament over recent months with insight into behavioural tendencies such as anxiety, fear, and aggression (16). In 2020, it was used with the dog-ADHD rating scale in a broad study looking at behavioural changes in dogs with IE pre- and post-IE diagnosis (6). This study followed a cross-sectional case–control design, where “healthy” control dogs were age and breed matched to epileptic dogs for comparison. Caregivers were to fill out each questionnaire once for the control dogs and twice for the epileptic dogs (retrospectively pre- and post-onset of epilepsy). First, the study showed that control dogs had overall higher trainability scores than dogs with IE, and dogs with IE had higher scores in dog-directed and non-social fear, aggression, attachment, and attention deficit (6). Interestingly, the results indicated that there was no relationship between medication status and any behavioural characteristic, which is inconsistent with other research findings (8, 22). This inconsistency regarding the impact of ASDs on behaviour warrants further investigation but is outside the scope of this review. Of note is that these behavioural questionnaires were previously validated and used to diagnose behavioural conditions in non-epileptic dogs, indicating their strength and relevance for behavioural work. Between the 2011 UK study and the more recent 2020 study, these validated questionnaire tools provide foundational evidence supporting the existence of behavioural comorbidities in canine epilepsy.

Evaluating a more precise time period with a bespoke questionnaire, a study in 2020 focused on caregiver-reported prodromal changes and seizure-precipitating factors in dogs with IE (21). Prodromal changes are long-term (hours to days) changes in disposition as an indicator of upcoming seizures, as defined by the International Veterinary Epilepsy Task Force (IVETF) (23). Seizure-precipitating factors are those events preceding a seizure that is accepted by the clinician as a reasonable trigger for the seizure (24). The goal of this study was to determine if caregivers recognize prodromal changes and seizure-precipitating factors and determine the caregivers’ perceived ability to predict seizures in their epileptic dogs (21). Caregivers of dogs with IE completed an online questionnaire regarding owner and canine demographics, epilepsy phenotype, use of ASDs, owner-perceived prediction abilities, and owner-perceived identification of prodromal signs and seizure precipitating factors (21). A total of 229 caregivers participated in the study, and all dogs were screened for IE using IVETF Tier I diagnostic criteria (1). The results of the study indicated that the three most common seizure-precipitating factors were changes in routine, stress, and overexertion, and the top three prodromal changes were clinginess, fear, and restlessness, as perceived by caregivers (21). The majority of caregivers (59.6%) felt they could predict an oncoming seizure. Of these caregivers, 28% felt they could predict an oncoming seizure within 5 minutes of seizure onset and 71.6% between 5 and 30 min of seizure onset (21). These results are an interesting starting point for investigating the minute changes in peri-seizure canine behaviour. Such results could be further validated using objective tools for behavioural analysis to compare against these caregiver reports of behaviour.

To further understand caregiver-reported phenomena that precede canine seizures and their time-frames, Forsgård and colleagues published a study investigating the prevalence of seizure-precipitating factors in dogs with IE (25). The researchers hypothesized that, as in people, seizure-precipitating factors are common in dogs with IE (25). Fifty dogs of various breeds and their caregivers were included in the study. Caregivers were asked 18 open-ended questions regarding signalment, personality, epilepsy-related factors, precipitating factors, and diagnosis history. Caregivers were also presented with a list of seizure-precipitating factors and were asked to check which ones applied to their dog. The mean age of participating dogs was 6.5 years, the mean seizure onset was 3.7 years, and the mean epilepsy duration was 2.8 years (25). The results of the study showed that 58% of caregivers reported their dog as having at least one seizure-precipitating factor. During the open-ended questionnaire, the most reported precipitating factors were stress, excitement, and hot weather (25). Interestingly, the checklist revealed the most common precipitating factors as visitors, changes in life and daily routine, disrupted sleep, new locations, and weather. Lastly, the majority of caregivers reported that seizures occurred within 24 h (35%) after the dog had been exposed to a precipitating factor, and 19% reported the seizure happened immediately after exposure (25). The difference in precipitating factors reported by caregivers with the different question formats highlights the diminished reliability of non-validated questionnaires when used without objective tools. This demonstrates the deep need to validate caregiver reports using objective tools that can confirm the presence of seizure-precipitating factors. Understanding seizure-precipitating factors in dogs will facilitate the exploration of the dog’s behaviour and environment peri-seizure to aid in seizure prediction.

#### 2.1.1. Limitations of questionnaires

Although questionnaires have provided researchers with an initial understanding of the prevalence of behavioural changes associated with various stages of canine epilepsy as per caregivers, users should be aware of potential limitations with the use of this tool. Firstly, questionnaires rely on owner reports, which can be prone to different types of bias such as social desirability bias where caregivers would feel pressure to provide a response they believe the researchers desire (26). These questionnaires were also collected retrospectively, subjecting the data to recall biases where caregivers may have simply forgotten details related to their dog’s behaviour and seizure history (27). Thus, the data provided by caregivers may not reflect accurate behavioural changes experienced by the dog. Lastly, relying on caregiver reports of seizure frequency and prodromal changes likely resulted in skewed data due to the tendency of caregivers to underreport seizure frequency as seizures can occur when the caregiver is not around, or the seizures are not outwardly visible (12). In subsequent research, these weaknesses could be improved upon by measuring canine behaviour using an objective behavioural tool such as Actigraphy and an objective seizure detection tool such as EEG, in addition to subjective caregiver questionnaires. Additionally, caregivers could complete these questionnaires in real-time to eliminate the presence of recall bias.

### 2.2. Electroencephalography

Electroencephalography (EEG) is the recording of the cerebral cortex’s electrical activity via electrodes on the skull, scalp, or surface of the cerebral cortex (28). Clinically accurate seizure diagnosis and categorization of seizure type in awake and behaving dogs can only be achieved using scalp EEG due to its sufficient spatial and temporal resolution (15, 29). Another form of scalp EEG, video-EEG (vEEG), combines cerebral cortex recordings with synchronized video of the dog to allow for confirmation of seizure events and visualization of ictal and interictal behaviours through both technologies. Historically, most canine epilepsy studies have obtained results using scalp EEG on sedated dogs, as sedation makes the instrumentation process significantly easier and reduces the risk of the dog removing the equipment during the recording (30–34). Although sedation reduces instrumentation and recording difficulties, it also eliminates the ability to analyze neuronal activity in naturally awake and behaving dogs. The ability to analyze the behaviour and neuronal activity of unsedated dogs is crucial to enhance the understanding of canine epilepsy and behaviour. Conversely, vEEG was able to identify the first reported case of absence epilepsy in a dog that initially presented with twitching and staring episodes by correlating these behaviours captured on video with absence seizure ictal patterns on the EEG recording (35). Thus, vEEG in unsedated dogs is a powerful tool that aids in accurate seizure diagnosis and categorization of seizure type.

vEEG has further benefits in pre-, post-, and interictal behavioural analysis. In 2017, James and colleagues were the first to investigate the relationship between vEEG recording length, frequency of reported seizure events prior to the recording, and diagnostic success in unsedated dogs (15). The researchers hypothesized that there would be a positive association between diagnostic success, increased recording length and increased frequency of reported seizure events (pre-recording) (15). A retrospective review of clinical cases revealed that vEEG confirmed the diagnosis of epileptic seizures in 43% of dogs through ictal and interictal discharges. Significantly, 57% of dogs displayed no EEG abnormalities despite the occurrence of a target event during their recording. Target events were behaviours the caregiver or clinician previously questioned whether they were ictal or non-ictal in nature (15). The results of the study also showed that as the frequency of previously reported events by the owner decreased, so did the likelihood the EEG would successfully provide a diagnosis. The researchers found no significant association between EEG recording length and diagnostic success, although a later larger study found an optimal recording length of greater than 4 hours (15). This study provides evidence to support that EEG is the only way to objectively confirm seizure activity directly through ictal discharges, or indirectly through interictal epileptogenic discharges. This means that cortical changes in electricity associated with seizure activity can be detected during seizures and between seizures (15). Additionally, this study highlights the effectiveness of vEEG to objectively confirm abnormal cortical discharges as seizure events while generating a synchronized video recording that allows for observation of seizure events and the behaviours that surround them. The objective confirmation of seizure events using vEEG helps to combat the seizure underreporting phenomenon in veterinary medicine, which in turn helps to reduce the risk of misinterpreting these events as abnormal behaviours rather than seizures (12). Thus, objective confirmation of seizures and the visualization of behaviours surrounding these seizures using vEEG can be used to aid in epilepsy diagnosis in a clinical and research setting until a simpler approach is developed for seizure detection through behavioural analysis.

Another behavioural application of vEEG for veterinary epilepsy studies is polysomnography. In polysomnography, vEEG in dogs correlates periods of rapid eye movement (REM) sleep and non-REM sleep with behaviours of interest (36). For example, REM sleep behaviour disorder, diagnosed using polysomnography, is characterized by excessive motor activity and/or abnormal behaviours experienced during REM sleep (36). Clinicians and researchers use EEG to correlate the cortical activity of REM sleep with behaviours such as chewing, biting, or barking to confirm REM sleep behaviour disorder diagnoses thus distinguishing these behaviours from sleep-associated seizures. Polysomnography has also provided insight into the relationship between sleep quality and cognition in dogs with canine cognitive dysfunction syndrome (CCDS) (37). Similar to people with Alzheimer’s disease, caregivers of dogs with CCDS often report that their dogs have difficulty sleeping. Using polysomnographic recordings and cognitive testing, CCDS dogs with higher dementia scores were found to spend less time in REM and NREM sleep during a two-hour window than dogs with lower dementia scores (37). This further highlights the efficacy of EEG for investigating sleep patterns in dogs with neurological diseases. Future studies could expand on this approach to evaluate polysomnography’s ability to detect changes in sleep in epileptic dogs.

Intracranial EEG (iEEG) is a more invasive form of EEG that requires surgical implantation of electrode strips in the subdural space to monitor cortical activity (38). The permanence of iEEG allows for long-term recordings without the concerns of losing electrodes and maintaining a low impedance (38). A 2011 study was the first to assess the feasibility of long-term continuous iEEG recordings in ambulating dogs and successfully collected 11,000+ hours of iEEG data including 202 seizures over a span of 5 months in six dogs with naturally-occurring epilepsy (38). A later study expanded on this approach by implementing classifiers that were trained to differentiate between pre-ictal and interictal iEEG output to forecast seizures (39). This group was able to detect 125 spontaneous seizures in 3 dogs with focal epilepsy over the span of 6.5 to 15 months (39). Further research investigating automatic seizure identification in iEEG has since evolved into subject-specific convolutional neural networks (CNNs) that are able to forecast seizures in real-time at a higher sensitivity than previously reported models (40). Most recently, iEEG has been used to identify seizure phase locking in dogs in which fluctuations of interictal spike rates that inform seizures reoccur at certain times in circadian and multiday cycles (41). Further, deep brain stimulation, a technique shown to interrupt epileptic networks and decrease interictal spikes, was able to modify these interictal spike fluctuations. Thus, iEEG proves itself a powerful tool for long-term data collection and shows promise in its preliminary ability to forecast seizures.

#### 2.2.1. Limitations of EEG

Although vEEG is extremely effective for capturing accurate seizure signalment and concurrent behaviours, some limitations exist. One limitation of EEG has been its practicality as a clinical tool, given its requirement for specialized training for the instrumentation and analysis process. Thus, implementing vEEG in routine clinical and research practices may prove difficult. Another limitation of scalp EEG is its inability to detect seizures of subcortical origin, which may skew seizure frequency counts and increase difficulty in locating the exact origin of the seizure (42). Thirdly, the success of utilizing vEEG in unsedated dogs is dependent on the dog’s temperament and level of tolerability, as some find discomfort in the handling and use of adhesive tape and equipment to secure the device and electrodes. Another limitation of vEEG is that classifying behaviours is tedious and time-consuming and relies on the caregiver or clinician to keep the video camera trained on the dog as much as possible. Additionally, vEEG is not suitable for long-term recordings, i.e., a week, due to battery life limitations. Although battery life has improved in recent years, dogs with infrequent seizures may not benefit from vEEG if events of interest are not captured during the recording as a negative recording does not rule out seizures or epilepsy. Despite its recent success, iEEG is significantly more invasive than vEEG and requires substantial commitment from owners due to extended surgical recovery, cost, and maintenance of external iEEG transmitter units (38–41).

### 2.3. Actigraphy

Actigraphy is a non-invasive technique that continuously measures a subject’s rest and activity levels. Accelerometers, devices used in actigraphy, measure static or dynamic accelerations of the body in multiple axes (38). In recent years, accelerometers have been used to identify normal behaviours and to detect abnormal health conditions in dogs such as obesity, osteoarthritis, and chronic pain (43, 44).

#### 2.3.1. Examination of canine behaviour

Collar-mounted actigraphy units need to be feasible for long-term wear in order to accurately understand canine behaviour. In 2018, Orymeyer and colleagues conducted a study using an Actigraph monitor and a PetPace collar to measure the physiological responses of dogs during different movement intensities, as well as the association between these physiological responses and the dog’s proximity to its caregiver (45). The PetPace collar measures activity level, pulse and respiration, and the Actigraph measures the dog’s proximity to its caregiver. The dogs wore the Actigraph unit and PetPace collar for 10–15 days, and the caregivers were asked to keep a log of all substantial interactions with the dog (e.g., nail trimming) (45). All 11 dogs were American Eskimos from a rescue facility. The data from the PetPace collar indicated that most of the dogs’ time was spent in a sedentary state (84%), and the amount of time in a sedentary state increased as age increased (45). The results also showed that as activity level increased from sedentary to vigorous, pulse and respiration also increased. Lastly, there was no significant difference between any physiological response at different proximities from the caregiver (45). One limitation of this study is that all the dogs included were the same breed and they were all rescues. Rescue dogs may have experienced physical abuse and neglect from previous caregivers, which negatively affects their perception of other caregivers until trust is established. This may have had an impact on the results of the proximity and physiological response portion of the study. This study provides useful insight into the feasibility of using wearable biomonitors on dogs for extended periods of time, as they successfully kept the Actigraph unit and PetPace collar on for a minimum of 10 days. Additionally, the Actigraph unit was successful in monitoring the dog’s movement intensities, which in future could be classified into different behaviours such as walking, running, or sleeping.

The recent development of canine movement algorithms for usage in accelerometry research has allowed for more specified behaviour classification. A study conducted in 2017 by Uijl and colleagues had a goal of using accelerometers to accurately classify eight different behavioural states in dogs in a clinical setting (46). These behavioural states were walking, trotting, cantering, inactive, sleeping, eating, drinking, and head shaking. Dogs were included in the study if they were deemed healthy and behaviourally sound by a veterinarian. Each dog (*n* = 51) was equipped with an accelerometer attached to their collar, and a video recording of the dog’s behaviour was synchronized to the data being obtained by the accelerometer. Certain behaviours were flagged on the video and accelerometer data was analyzed to see if these behaviours were captured. The results of the study showed that the probability of the accelerometer detecting a behaviour event that matched with the video event ranged from 0.93 (sleep) to 1.00 (trot) (46). In addition, the probability that the accelerometer did not detect a behaviour event that did not actually occur ranged from 0.85 to 1.0. Lastly, the proportion of all behavioural events that were classified correctly was above 95% for all states except for inactive (91%) and sleep (94%) (46). This study is one of the first to successfully validate the use of an accelerometer for identifying specific behavioural states in dogs. The authors of this study had a clear and concise definition for each behavioural state which will be useful for prospective studies examining canine behaviour. These validated behavioural classifications using accelerometer data could be useful in an investigation of the behaviours exhibited by epileptic dogs in the peri-seizure period. Future investigations could supplement accelerometers with EEG to allow for more accurate classification of resting states such as sleep and inactivity.

#### 2.3.2. Examination of canine seizures

Recently, accelerometry has been introduced into veterinary epileptology with the aim of identifying seizure activity. A study conducted in 2020 investigated the effectiveness of using accelerometers to detect generalized tonic–clonic (GTC) seizures in dogs with epilepsy (18). The dogs included in the study were given an accelerometer to wear on their collar for the duration of the six-month study. The caregivers were instructed to keep a detailed log of all seizure activity, including date, time, and seizure characteristics (18). The caregiver-reported seizure activity was time-matched to a pre-determined algorithm for the accelerometer to detect and confirm seizure activity. In total, 19 dogs were included and 215 GTC seizures were documented by caregivers over the six-month study period. When compared to caregiver-reported seizures, 70 seizures were detected successfully and 278 seizures were falsely detected using the accelerometer (18). The authors concluded that GTC seizures in dogs can be identified using an accelerometer at a low sensitivity and were unsuccessful in detecting other seizure types such as absence or focal.

In 2022, another study investigated the feasibility of accelerometers for GTC seizure detection through the development and trial of algorithms that classified accelerometer data as GTC seizure activity or non-seizure activity (47). Four epileptic dogs were monitored with accelerometers for up to 78 h, and three GTC seizures were accurately identified using the developed algorithm. These results provided further evidence to support the feasibility and accuracy of accelerometers for identifying GTC seizures in dogs.

#### 2.3.3. Limitations of actigraphy

Despite the growing recognition and improvement of accelerometers in canine veterinary research, multiple limitations to their usage exist. Firstly, an agreement has yet to be reached regarding the optimal placement of these sensors on dogs. Some studies suggest that placement on the collar is most effective and provides more accurate data, whereas others state that placement on the dog’s back is more effective (46, 48). Additionally, accelerometers concurrently measure acceleration and gravity which becomes difficult when attitude from gravity (i.e., the unit’s orientation and position) is not consistent, e.g., when the dog is not located on flat ground, as the unit requires a fixed and stable position to retain accurate measurements (48). Thus, updated accelerometers need to be developed and validated in dogs to account for changes in attitude. Secondly, the sole use of accelerometers is not sufficient for detecting all ictal events due to their inability to capture non-GTC seizures such as absence and focal seizures. Non-GTC seizures are significantly less physically jarring in nature and often present as minor movements such as facial twitching, excessive blinking and lip licking and can be specific to the individual dog. Using only accelerometers to identify and classify seizures may result in a significant number of seizures going undetected (18). To detect seizure events accurately accelerometers would need to be combined with technology that can detect non-GTC seizures, such as EEG, at least for training detection algorithms. This further highlights the importance of EEG as the reference standard in the diagnosis of epilepsy in dogs, as EEG detects GTC and non-GTC seizures at a high sensitivity. Lastly, behaviour misclassifications can occur due to noise captured in the accelerometer signal. Noise may be a result of multiple behaviours occurring at once, or interruptions from other physiological systems (49).

## 3. Discussion

Questionnaires, EEG, and actigraphy have all successfully contributed to the understanding of seizures and behavioural contexts in canine epilepsy. Although useful, each of these tools has limitations that hinder researchers’ ability to capture an accurate, complete, and objective picture of behavioural comorbidities in canine epilepsy. Questionnaires have provided researchers with behavioural changes caregivers have reported in their epileptic dogs from pre- to post-diagnosis, as well as prodromal changes in behaviour leading up to a seizure. Thus, questionnaires allow researchers to investigate changes in seizure characteristics and behaviour granularly and broadly across time depending on their research goals. The main limitation of the behavioural data collected from these questionnaires is that it comprises caregiver-reported, subjective accounts of behaviour. Retrospective accounts are prone to recall bias, as they recount behavioural and seizure contexts from the distant past (months to years). Ideally, capturing and quantifying behaviour objectively and in real-time or prospectively in addition to caregiver-reported questionnaires would provide the most accurate behaviour data for canine epilepsy studies. EEG remains the gold standard for seizure detection and classification in canine epilepsy, as it is the only way to definitively confirm seizure activity directly (ictal discharges), or indirectly (interictal epileptogenic discharges). The use of vEEG could be a useful tool for investigating behavioural changes in epileptic dogs on a more granular scale, such as pre-and post-seizure, but is limited to the length of the recording and may pose a privacy concern for some caregivers. Unfortunately, most research utilizing EEG in dogs thus far has only collected data on sedated subjects and therefore has not been able to collect accurate behavioural data in a home environment. The use of vEEG in awake and behaving dogs could allow researchers to visualize behaviours as they occur and establish a temporal relationship between these behaviours and ictal events. Although actigraphy has had mixed results in terms of seizure classification, it has been successful in classifying behavioural states in dogs and can be useful as an objective behavioural assessment method to supplement owner reports. Additionally, behaviour can be examined over relatively long periods of time (weeks to months) due to improved battery function of devices in recent years. Future research could investigate these behavioural states in different canine populations (i.e., diseased) to determine whether there are consistent behavioural differences between groups for further development of diagnostic and treatment monitoring tools.

### 3.1. Potential developments

Prospective investigations could expand on and validate current canine behavioural research methods by combining vEEG, actigraphy, and caregiver-reported questionnaires in awake and behaving dogs to obtain a complete and accurate assessment of epileptic dog behaviour. vEEG could be used to objectively determine seizure frequency and provide visualization of behavioural changes that may occur ictally or interictally such as pacing or panting, as confirmed by EEG output. Actigraphy could be used to assess time spent in different behavioural states, proximity to the caregiver and other key locations in the environment to track what the dog is doing objectively. Actigraphy data can also be compared against EEG data to determine if changes in the ictal state correlate to changes in behaviour. To aid in actigraphy and EEG analysis, algorithms or artificial intelligence techniques could be applied to the data to improve behaviour and seizure classification definitions. Questionnaires could be used to gain a detailed understanding of the dog’s seizure characteristics, as well as their typical housing and behaviour to understand general trends and potential triggers for seizure activity. The data obtained from these questionnaires could act as a baseline for comparisons between normal, pre-, and post-seizure behaviour in a single dog. Another method for providing baseline data would be to compare data collected from all three tools between neurotypical and epileptic dogs. The two cohorts could be age- and breed-matched to help ensure that data between groups is sufficient for comparison. Various lengths of vEEG and recording time could be used depending on hardware limitations (e.g., battery life), caregiver cooperation, and the dog’s temperament. Ideally, recordings would extend at least 24 h in order to capture an accurate representation of the dog’s typical behaviour cycle. The longer the recording, the greater the chance of capturing one of the dog’s seizures which would allow for comparisons of behaviour pre- and post-seizure. Thus, simultaneously utilizing vEEG, Actigraphy, and caregiver-reported questionnaires in awake and behaving dogs for long periods could act as an important step towards finding a method to objectively analyze behaviour in epileptic dogs.

Capitalizing on technological advances to increase our understanding of epileptic canine behaviour is crucial for improving treatment options in dogs with epilepsy given that ASD therapy for dogs is inexact, with considerable drug resistance or adverse effects that affect the quality of life (9). Identifying techniques for seizure prediction will positively impact the dog and caregiver’s quality of life, which is affected by the emotional experience of working with the seizing animal and the time and financial commitments required for seizure management. The concurrent use of EEG and actigraphy would allow for a better understanding of more granular (hours to days) behavioural changes surrounding canine seizures. Additionally, this combination of technology would aid in the ability to distinguish epileptic psychosocial comorbidities from other causes of abnormal behaviour such as ASD adverse effects, focal and absence seizures, interictal, and pre and postictal behaviours. This combination of technology with the addition of previously validated caregiver-reported questionnaires would allow for a better understanding of the behavioural and environmental context of canine seizures at various time granularities depending on the questionnaire used. Through this understanding, caregivers and clinicians may be able to predict oncoming seizures and treat them accordingly in hopes of decreasing seizure frequency and improving the dog and caregiver’s QoL.

## 4. Conclusion

Behavioural comorbidities in dogs with epilepsy have become increasingly recognized in veterinary medicine. Previous investigations of these comorbidities use subjective measures such as caregiver-reported questionnaires without a tool to objectively assess epileptic canine behaviour. A promising approach for the objective assessment of canine behaviour could consist of simultaneous vEEG and Actigraphy recordings in awake and behaving epileptic dogs, in addition to caregiver-reported questionnaires. vEEG would provide confirmation of ictal state and seizure frequency, Actigraphy would provide the behavioural context in correlation with EEG output, and questionnaires would provide a detailed description of seizures, behaviours, and the dog’s environment. The combination of these tools would allow for a thorough understanding of the behavioural and environmental context of canine seizures. This knowledge could then be used by caregivers and clinicians in a personalized medical context to potentially predict oncoming seizures and treat accordingly in hopes of decreasing seizure frequency. Thus, future research would be improved by technologies that objectively assess canine behaviour to allow for a better understanding of behavioural comorbidities in dogs with epilepsy as a crucial step towards seizure prediction.

## Author contributions

EF, LN, and FJ: conception. EF and FJ: drafting the review. EF, LN, LG, and FJ: revising the article for intellectual content. All authors contributed to the article and approved the submitted version.
